# Sequential Changes in Circulating Tumor Cells in the Peripheral Blood of Pancreatic Cancer Patients with Preoperative Chemotherapy Using a New Immunocytology-Based, Light Microscopic CTC Detection Platform

**DOI:** 10.3390/diagnostics15060752

**Published:** 2025-03-17

**Authors:** Kohei Yasui, Takuya Saito, Sho Ueda, Kentaro Shinohara, Yasuyuki Fukami, Tsuyoshi Sano, Hayao Nakanishi

**Affiliations:** 1 Department of Gastroenterological Surgery, Graduate School of Medicine, Aichi Medical University, Nagoya 480-1195, Japan; yasui.kouhei.891@mail.aichi-med-u.ac.jp (K.Y.); ueda.shou.106@mail.aichi-med-u.ac.jp (S.U.); shinohara.kentarou.857@mail.aichi-med-u.ac.jp (K.S.); fukami.yasuyuki.279@mail.aichi-med-u.ac.jp (Y.F.); sano.tsuyoshi.508@mail.aichi-med-u.ac.jp (T.S.); 4444hn@gmail.com (H.N.); 2 Laboratory of Clinical Pathology, Okazaki City Hospital, Okazaki 444-0002, Japan

**Keywords:** circulating tumor cells (CTCs), cytology-based CTC detection, preoperatve chemotherapy, pancreatic cancer, therapeutic marker

## Abstract

**Background:** Circulating tumor cells (CTCs) have recently been developed as biomarkers. Several studies have reported on the clinical use of CTCs to assess drug resistance in various cancers. However, sequential and multiple CTC measurements during chemotherapy are relatively rare. We recently reported a transient increase in CTCs early after chemotherapy by sequentially detecting CTCs in a human pancreatic cancer xenograft model in nude mice. **Method**: In the present study, using a newly developed immunocytology and glass slide-based convenient CTC detection platform, we examined CTC numbers sequentially before, during, and after chemotherapy in the peripheral blood of 14 pancreatic cancer patients, pathological stage (pStage) I-IV, who underwent surgery with preoperative chemotherapy and GS (Gem/S-1) and GnP (Gem/nab-PTX). **Results:** Among patients with strongly or weakly elevated CTC counts (3–44/5 mL of blood) following GS treatment, four out of six pancreatic cancer patients were judged to have a partial response (PR), and two out of six were deemed to have stable disease (SD) as a clinical response based on the CT image. In contrast, in patients with GnP therapy, three out of four patients showed no CTC response, and these three patients were judged to have progressive disease (PD), while the remaining one patient was judged to have SD in terms of their clinical response. **Conclusion**: These results suggest that sequential CTC monitoring during preoperative chemotherapy in pancreatic cancer patients can be a helpful liquid biopsy diagnostic tool as a therapeutic marker to predict tumor chemosensitivity and chemoresistance in clinical settings. Further large-scale clinical studies are required to confirm and clarify this hypothesis.

## 1. Introduction

Pancreatic cancer (PC) ranks as the sixth leading cause of cancer-related mortality worldwide [1]. Surgery remains the only curative treatment modality available for achieving long-term survival in PC patients; however, the median survival of those undergoing radical pancreatectomy alone is approximately 22 months, with a reported 5-year survival rate of 12.2% [2]. Chemotherapy, including adjuvant and neoadjuvant therapy, improves the median and 5-year overall survival (OS) of patients with curatively resected pancreatic cancer [3,4]. Neoadjuvant chemotherapy (NAC), utilizing agents such as FOLFIRINOX, GEM monotherapy, GEM/nab-PTX, and GEM/S-1, offers several advantages over upfront surgery. These benefits include the delivery of systemic chemotherapy to nearly all surgical candidates and an improved overall survival (OS) due to a higher rate of negative-margin resection [5,6]. Despite advancements in treatment strategies, the prognosis of pancreatic cancer patients still remains poor. This is primarily attributed to the challenges in early imaging-based diagnosis and the difficulty of gaining a timely assessment of drug sensitivity and resistance during chemotherapy [2].

Circulating tumor cells (CTCs) in peripheral blood have long been regarded as potential biomarkers that can give diagnostic and therapeutic solutions for various types of cancers. CTCs in the blood offer minimally invasive and reproducible specimens for detecting tumor cells during multiple types of therapies [7,8,9,10]. Many clinical studies on pancreatic cancer have revealed that increased numbers of CTCs are associated with tumor progression and are correlated with short survival, indicating the utility of CTCs as prognostic markers [7,8]. In addition, several studies have reported the clinical use of CTCs to assess drug susceptibility/resistance in various types of cancers [9,10,11]. However, their response to various types of cancer therapies still remains somewhat unclear. We recently reported that CTCs increase transiently and then decrease shortly after chemotherapy in human breast and pancreatic cancer xenograft mouse models [12,13]. The histological analysis of mouse primary tumor tissue revealed that the peak of transiently increased CTC after chemotherapy coincided in time with the maximal mitotic arrest of tumor cells in the primary tumor. This suggests the possibility that the transient increase in CTCs shortly after chemotherapy is the result of passive tumor cell mobilization into the blood due to severe lysis and the destruction of primary tumor tissues by chemotherapy [13]. Therefore, sequential and multiple enumerations of CTC numbers during chemotherapy may be a potential indicator of chemotherapeutic responses in the clinical setting.

To date, most CTC detection platforms use a multicolor immunofluorescence staining method, such as Keratin+/EpCAM+/CD45−/DAPI+ [14,15]. However, reports on immunocytology-based CTC detection methods are limited [16]. Recently, we developed a new and convenient CTC detection platform using filtration and an immunocytology-based CTC detection system under a light microscope [17,18,19]. Using this convenient immunocytology-based platform, we conducted a pilot clinical study with 12 patients with pancreatic cancer who underwent preoperative chemotherapy to understand the dynamic aspects of CTCs and the importance of sequential CTC enumeration during chemotherapy in clinical settings.

## 2. Materials and Methods

### 2.1. Reagents

A mouse monoclonal antibody (Clone, Oscar) against human-wide-spectrum (pan)-cytokeratin was obtained from BioLegend (Dedham, MA, USA) for the detection of CTCs. Immunostaining and subsequent color development were conducted using the DAKO system, as described below. For nuclear counterstaining, Meyer’s hematoxylin was also used.

### 2.2. Patients and Bloods

Patients with pStage I–IV primary pancreatic cancer (*n* = 14) who underwent surgery at Aichi Medical University Hospital between 2023 and 2024, were enrolled in this study. The average age of the patients was 72 years, and the male/female ratio was 7/7. Peripheral blood (5–6 mL) was collected from the cubital vein. The punctured blood samples were pooled in specialized tubes for liquid biopsy (Streck, La Vista, NE, USA) and kept at room temperature, and CTCs were collected within 24 h after puncture using our original platform. The tumors ranged from stage I to IV, and their histology was adenocarcinoma based on the UICC (Union for International Cancer Control) criteria (Table 1). This study was approved (approval number: 2022-166) by the Institutional Ethics Review Board of Aichi Medical University Hospital. Before sample collection, written informed consent was obtained from each patient. This study meets the standards defined by the principles outlined in the Declaration of Helsinki.

### 2.3. Filtration and Slide Glass-Based CTC Detection

**Figure 1:**
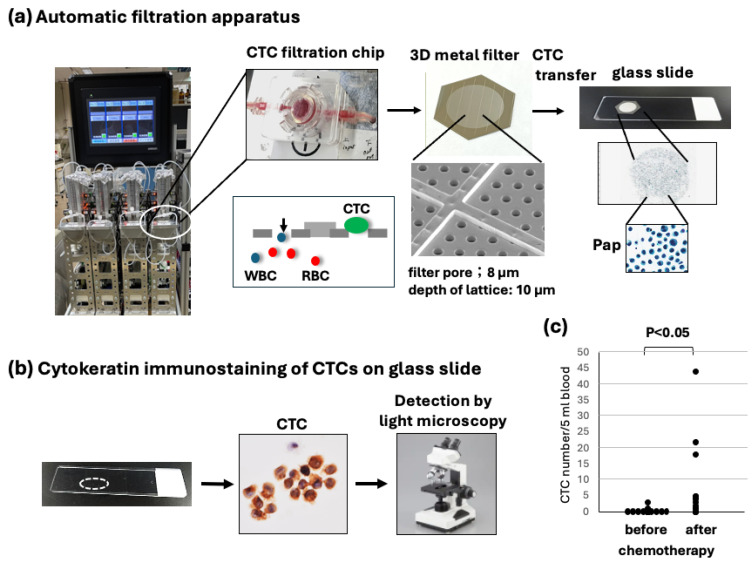
Overview of immunocytology-based circulating tumor cell (CTC) detection platform using filtration and subsequent transferring of CTCs to a glass slide. (**a**) After collecting CTCs on the 3D metal filter in the automated CTC enrichment apparatus, the filter is placed upside down on a glass slide, and CTCs are transferred to a glass slide by air-pressure-mediated transfer apparatus. Finally, CTC slides are immediately fixed and stored in 95% ethanol at 4 °C until staining. (**b**) CTCs on a glass slide are stained by keratin immuno-cytochemistry, and a brown color is developed. Resultant keratin-positive cells with hematoxylin-stained atypical large nuclei are judged as CTCs and are counted under light microscopy. (**c**) Comparison of the CTC number before and after chemotherapy (max number). CTC numbers after chemotherapy vary greatly but are significantly higher than CTC numbers before chemotherapy (*p* < 0.05).

Filtration-based microfluidic chips (polycarbonate polymer) containing a three-dimensional (3D) nickel filter with 8 µm pores and a 10 μm thick lattice structure were originally produced by Optnics Precision Co., Ltd. (Tochigi, Japan). An automated CTC collection apparatus with a fluid pressure control system and the abovementioned filter chip for the collection of CTCs and subsequent immunocytology-based detection of CTCs was originally produced by Maruyasu Industries Co., Ltd. (Okazaki, Japan) (Figure 1). This automated CTC enrichment apparatus is equipped with four metal filter chips to run four blood samples simultaneously.

After automated filtration of the diluted blood samples and washing with PBS containing 5 mM ethylenediaminetetraacetic acid (EDTA), the CTCs on the filter were fixed in the chip with 10% buffered formalin for 30 min, followed by washing with PBS/EDTA. The 3D metal filter was detached from the microfluidic chips and placed upside down on a coated glass slide (MAS coat; Matsunami, Osaka, Japan). CTCs were transferred without damage and fixed to a coated glass slide using an air pressure-mediated original transfer apparatus (Maruyasu Industries Co., Ltd.). The recovery rate of CTCs from the filter on the glass slide was at least 75%, which was measured by the spike experiment using 100 cultured Suit-2 human pancreatic adenocarcinoma cells in 5 mL of blood. The resultant glass slide containing fixed CTCs (CTC glass slide) was immediately immersed and stored in 95% ethanol at 4 °C for subsequent immunostaining (Figure 1). The preservation of the CTC glass slide in cold ethanol is possible for at least 3–4 weeks.

### 2.4. Immunocytochemistry Using CTC Glass Slide Specimens

The immunocytochemistry of CTCs on glass slides using cytokeratin antibodies was performed as follows: after treatment with peroxidase blocking (for HRP) and a subsequent protein-blocking reagent, the CTC specimens were incubated with a mouse monoclonal anti-pan cytokeratin antibody (Oscar) for 40 min. After washing using the DAKO buffer, the specimens were incubated with an HRP-labeled polymer-conjugated goat anti-mouse antibody (EnVision + system) (DAKO, Carpinteria, CA, USA) for 20 min. After washing, a brown color was developed using a liquid DAB + substrate (DAKO). Nuclei were counterstained with Meyer’s hematoxylin. For counting cytokeratin-positive CTC numbers and estimating CTC morphology, the CTC glass slide was observed under a light microscope (Olympus BX50, Tokyo, Japan) by pathologists or cytologists. It is of note that the pan-cytokeratin antibody is known to stain almost all types of carcinoma cells as well as spindle-shaped carcinoma cells with sarcomatous changes or vimentin-positive epithelial–mesenchymal transition.

### 2.5. Statistical Analysis

The significance of differences between the two groups was determined using Student’s *t*-test and Wilcoxon’s signed-rank test. A *p*-value of less than 0.05 (*p* < 0.05) was judged as significant.

## 3. Results

### 3.1. Sequential CTC Detection Using Immunocytology-Based Platform Under Microscopy

After the collection and fixation of CTCs by metal filtration and their subsequent transfer to glass slides, fixed CTCs on glass slides were stained by immunocytology using a pan-cytokeratin antibody and HRP-labeled secondary antibody, followed by color (brown) development (Figure 1a,b).

**Figure 2:**
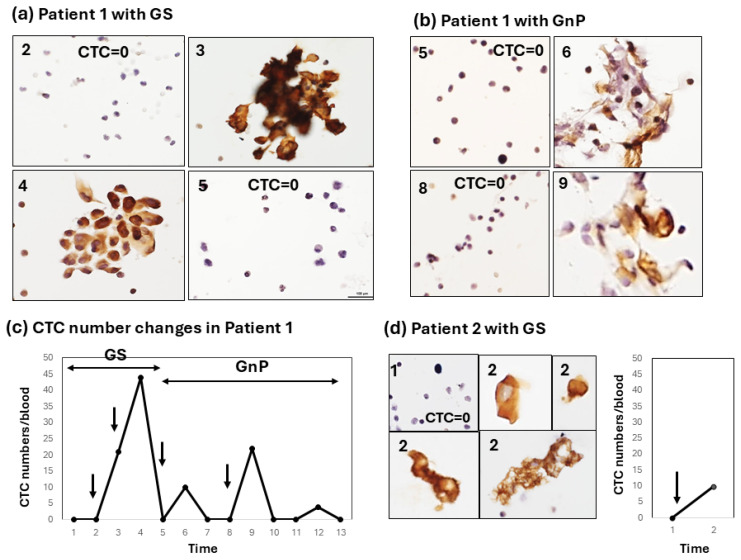
Type-1 CTC response showing a strong increase early after chemotherapy. (**a**) Sequential changes in the CTCs of patient 1 after chemotherapy with GS. Large cluster-type CTCs are seen. Numbers (2, 3, 4, 5) in the left-upper corner of the figure correspond to the number located in the horizontal axis (Time) of the line graph; (**b**) Sequential changes in the CTCs of patient 1 after chemotherapy with GnP. Cluster-type CTCs with some degenerative changes are seen (6, 9); Numbers (2, 3, 4, 5, 6, 8, 9) in the left-upper corner of the Figure 2a,b correspond to the number located in the horizontal axis (Time) of the line graph in Figure 2c. (**c**) The line graph shows sequential changes in the CTC number throughout combination chemotherapy with GS and a subsequent GnP regimen; (**d**) Changes in the CTCs of patient 2 after chemotherapy with GS. Single-cell CTC and small-clustered CTCs with some degenerative changes are seen (2). In patient 2, GS therapy stopped after only one round because of the patient’s worsening symptoms. Arrows in the line graph in Figure 1c,d indicate timing of chemotherapy.

**Figure 3:**
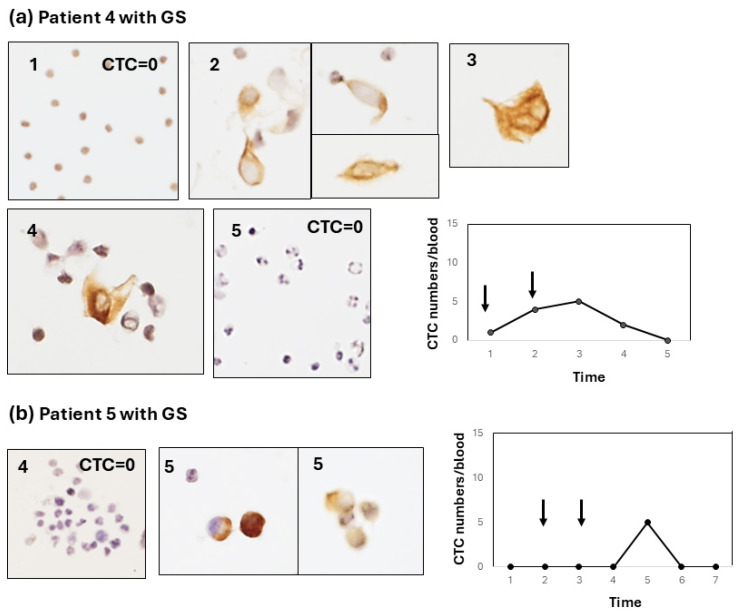
Type-2 CTC response showing a weak increase in the early-to-late stage after chemotherapy. (**a**) Sequential changes in the CTCs of patient 4 after chemotherapy with GS. Most of the CTCs are single cells with some small clusters. The line graph shows a mild increase in the CTC number throughout chemotherapy with GS (arrows). The CTC number gradually and weakly increased (<5 cells/blood) after GS therapy and decreased thereafter. (**b**) Sequential changes in the CTCs of patient 5 after chemotherapy with GS. Like patient 4, most CTCs are single cells. The line graph shows a weak increase late after chemotherapy with GS.

**Figure 4:**
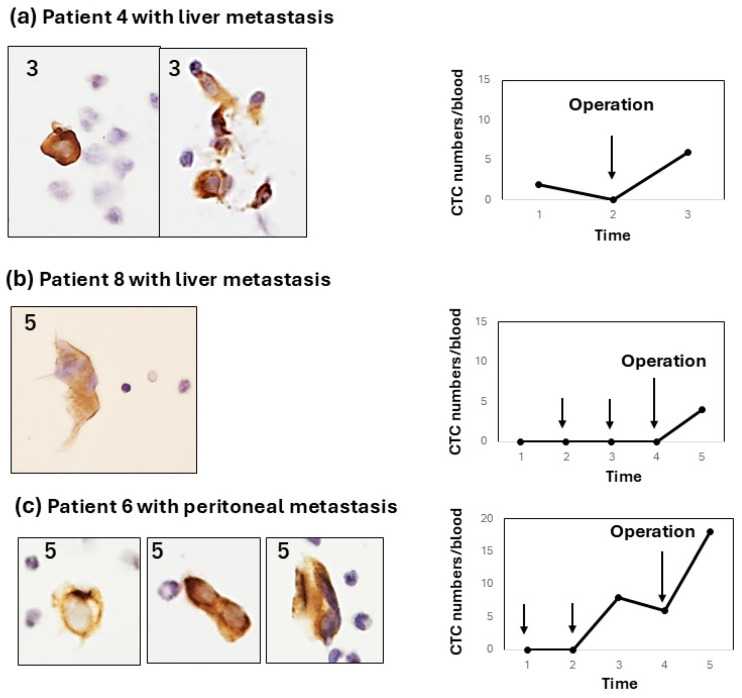
Type-3 CTC response showing a weak increase after pancreatectomy or exploratory laparotomy. All type-3 responses occur in patients with liver or peritoneal metastasis. (**a**) In patient 4 with liver metastasis, CTC changes occurred after exploratory laparotomy; (**b**) In patient 8 with liver metastasis, CTC changes only occurred after pancreatectomy; (**c**) In patient 6 with peritoneal metastasis, CTC increased after chemotherapy with GS and further increased after pancreatectomy. In all cases, CTCs were mostly single cells with some small clusters.

This method of CTC enumeration on glass slides under light microscopy seems to be more accurate and convenient than the fluorescence detection of CTCs under a dark field. In this system, the CTC specimen was permanent, and the time used for counting CTCs was within 5–10 min, including photography. It is advantageous that CTCs with degenerative change after chemotherapy could be morphologically estimated (Figure 2, Figure 3 and Figure 4).

### 3.2. Patient Characteristics

Fourteen patients with pancreatic cancer who received surgery at Aichi Medical University Hospital were included in this study. The clinical and pathological characteristics of the patients are summarized in Table 1. Briefly, the use of NAC therapy with GS and GnP was 50% and 29%, respectively, and the pStage of pancreatic cancer ranged from I to IV, and the histology was all adenocarcinoma.

### 3.3. Enumeration of CTCs Before, During, and After Therapy in Pancreatic Cancer Patients

The CTC number was first compared before and after chemotherapy (max CTC number). The number of CTCs clearly increased during chemotherapy compared to the number before chemotherapy at statistical significance (*p* < 0.05, *n* = 12) (Figure 1c).

The patterns of this increase in CTC numbers after chemotherapy can be classified into three types. Type 1: a moderate-to-strong increase in CTC numbers in the early period (1–2 weeks) after chemotherapy. In patient 1, for example, combination chemotherapy with the first GS followed by the GnP regimen was administered (Figure 2a–c). In the first chemotherapy session with GS, the CTC number strongly increased to a maximum of 44 cells/5 mL with large CTC clusters and decreased to 0 within 2 weeks after chemotherapy (Figure 2a). In patient 1, chemotherapy was then changed to three courses of the GNP regimen. CTCs increased each time 1–2 weeks after one, two, and three courses of the GnP regimen (Figure 2b,c). However, the increase in the CTC number after GnP therapy appeared to be weaker than that after GS chemotherapy, and the CTC morphology appeared to be somewhat degenerative. In patient 2, the disease condition was so critical that only single-shot GS therapy was possible. A moderate increase in the number of CTCs (10 cells/5 mL) was observed shortly after GS therapy. CTC clusters with a degenerative change similar to those observed in patient 1 were also noted in this case. (Figure 2d).

Type 2: the CTC change shows a weak increase (<10 cells/5 mL) in CTC numbers from the early-to-late period after chemotherapy. For example, this type of CTC change was shown in two patients (patient 4, Figure 3a; patient 5, Figure 3b). In patient 4, the CTC weakly and gradually increased and decreased. In contrast, patient 5 showed a weak, sharp increase late after chemotherapy. In these type-2 cases, CTCs were observed as single cells or small-cell clusters. Type 3: the CTC increases after surgery (pancreatectomy or exploratory laparotomy), irrespective of the increase in the CTC number during preoperative chemotherapy. Interestingly, two out of three patients (patients 4 and 8) with a postoperative CTC increase showed liver metastasis, and one patient (patient 6) showed peritoneal metastasis.

These results are summarized in Table 2. Of the eight patients who received GS chemotherapy, two patients showed a type-1 (early/strong) response (2/8), and five patients showed a type-2 (early to late/weak) response (5/8) to chemotherapy. One patient showed no response to chemotherapy. In particular, in patient 1, a strong increase was observed early after chemotherapy, irrespective of the GS and GnP regimens. The clinical effects of chemotherapy were categorized as a partial response (PR), stable disease (SD), and progressive disease (PD) based on CT image diagnostics. The clinical effect could not be determined (ND) in patient 2 because chemotherapy was stopped after only one shot because of the worsening of the patient’s condition.

In contrast, in four patients who received chemotherapy with the GnP regimen, three out of four patients showed no significant increase in CTC, and only one patient (patient 10) showed a late/weak CTC response. This finding is consistent with the clinical effects of chemotherapy. In fact, four out of four of the patients were evaluated as having progressive disease (PD), and one out of four patients had stable disease (SD). This result may be partly due to the short GnP chemotherapy period compared to the oral agent GS, which may have relatively high drug tolerability.

## 4. Discussion

We recently developed a unique immunocytology-based CTC detection platform that made it possible to conveniently and sequentially enumerate CTCs under light microscopy and for the mutation analysis of CTCs by extracting DNA from CTCs fixed on glass slides [19]. The key technologies of our platform are as follows: (1) the collection of CTCs using a 3D metal filter chip and a fluid pressure control system with four samples simultaneously; (2) subsequent CTC transfer from the filter to the glass slide by an air pressure-mediated CTC transfer device without damage; and (3) glass slide-based immunocytological detection using a pan-cytokeratin antibody under a microscope. To our knowledge, this CTC detection platform is the first device to enable the correct, convenient (within 3 h), and low-cost detection of CTCs, which is critical for the sequential detection of CTCs in clinical settings in hospitals. In the present study, using this platform, we successfully conducted a sequential enumeration of CTCs before, throughout, and after preoperative chemotherapy in clinical settings for the first time.

In this small-scale clinical pilot study, we obtained the following interesting results. The number of CTCs sharply increased in two patients and weakly increased in three patients in response to preoperative chemotherapy with GS in this clinical study. The first two patients correspond to patients with a type-1 response, and the next three patients had a type-2 response to chemotherapy. Patients with a type-2, weak CTC response seemed to differ from the response observed in the mouse xenograft model. Consequently, only two pancreatic cancer patients with a type-1 response to GS therapy corresponded to the human pancreatic cancer xenograft mouse model treated with FFX and GnP [13]. Unlike the pancreatic cancer xenograft mouse model, CTC responses to chemotherapy in clinical studies are greatly influenced by the disease stage, chemotherapeutic regimen/interval/length, the chemotherapeutic responsiveness of individual tumors/patients, and the patient’s disease condition [20,21]. Although there were significant variations in CTC responses in the clinical study, we could confirm a clear transient increase in the CTC number after chemotherapy, even in this small-scale clinical study. In addition, we observed CTC clusters and clusters with some degenerative changes (Figure 2a,b,d), which is similar to the CTCs in the mouse xenograft model. The presence of CTC clusters is reportedly known to promote chemotherapy evasion and metastatic seeding [22]; therefore, the presence of large-cluster-type CTC may also have prognostic/predictive value.

In conclusion, to the best of our knowledge, this study is the first to demonstrate that CTCs transiently increase after effective preoperative GS chemotherapy, at least in part in pancreatic cancer patients with a type-1 response to chemotherapy. This suggests that sequential CTC enumeration is a potential predictor of chemosensitivity/resistance. To date, however, such a transient increase in the CTC number after chemotherapy has not been well recognized. At present, it is well known that an increase in CTCs after chemotherapy is associated with drug resistance, whereas a decrease in CTCs suggests that treatment is effective [10,23]. So, there is some discrepancy in the interpretation of CTCs observed after chemotherapy among the studies described above. To clearly understand the CTC–drug sensitivity relationship, further large-scale clinical studies with sequential and multiple CTC enumerations are required in the near future.

Recent studies have reported on predictive markers for chemotherapy in cancer patients, including circulating tumor DNA and patient-derived organoids. However, these indicators are expensive and time-consuming and, therefore, are not practical in hospital settings [24,25,26]. In this study, in the longest case, the CTC detection period spanned 6 months with 13 tests. This seems to be a heavy burden on patients. However, the sequential enumeration of CTCs with 5 mL of blood collection every 2 weeks during chemotherapy can be an acceptable diagnostic tool to predict tumor chemosensitivity/resistance and risk for metastasis in the clinical study. Another potential clinical application of CTC detection during chemotherapy is genetic analysis with an increased number of CTCs that are usually difficult to obtain from the peripheral blood of patients who do not receive chemotherapy [27]. A further large-scale clinical study for sequential CTC enumeration before, during, and after chemotherapy is warranted in the near future.

## Figures and Tables

**Table 1:** Clinical characteristics of pancreatic cancer patients.

Parameters		Number (*n* = 14)
Age	Median (range)	72 (52–86)
Sex	Male	7 (50%)
	Female	7 (50%)
Location	Head	8 (57%)
	Body	4 (29%)
	Tail	2 (14%)
Neoadjuvant chemotherapy (NAC)	GS	7 (50%)
	GnP	4 (29%)
	GS→GnP	1 (7%)
	None	2 (14%)
Tumor size	T1	2 (14%)
	T2	3 (21%)
	T3	9 (64%)
Lymph node metastasis	N0	12 (86%)
	N1	2 (14%)
Pathological stage (pStage)	I	1 (7%)
	II	5 (36%)
	III	0 (0%)
	IV	8 (57%)
Histology	Adenocarcinoma	14 (100%)
	Others	0 (0%)
Clinical effect of chemotherapy	PR	5 (36%)
	SD	3 (21%)
	PD	3 (21%)
	ND	3 (21%)

PR, partial response; SD, stable disease; PD, progressive disease. ND, not determined.

**Table 2:** Types of CTC increase in response to chemotherapy and its relation to several clinico-pathological factors.

Patient	Regime	Type (Response to Chemotherapy)	Clinical Effect of Chemotherapy	pStage	Metastasis	Post Operative CTC
1	GS + GnP	Type 1 (Early/strong)	PR	T3N0M1	Peritoneum	Negative
2	GS	Type 1 (Early/moderate)	ND	T3N0M1	Liver	Negative
3	GS	Type 2 (Early/weak)	SD	T1N1M0		Negative
4	GS	Type 2 (Early/weak) + Type 3	PR	T3N0M1	Liver	Positive
5	GS	Type 2 (Late/weak)	PR	T3N0M0		Negative
6	GS	Type 2 (Late/weak) + Type 3	PR	T3N0M1	Peritoneum	Positive
7	GS	Type 2 (Late/weak)	SD	T3N0M0		Negative
8	GS	Type 3 (No response)	PR	T3N0M1	Liver	Positive
9	GnP	No response	PD	T3N0M1	Lung	Negative
10	GnP	Late/weak	PD	T3N0M0		Negative
11	GnP	No response	PD	T2N0M1	Peritoneum	Negative
12	GnP	No response	SD	T2N0M0		Negative
13	None	ND	ND	T1N0M0		Negative
14	None	ND	ND	T2N1M0		Negative

PR, partial response; SD, stable disease; PD, progressive disease. ND, not determined.

## Data Availability

The original contributors presented in this study are included in the article; further inquiries can be directed to the corresponding author.

## Abbreviations

The following abbreviations are used in this manuscript:

CTC	Circulating tumor cells
PC	Pancreatic cancer
